# Supplementary material for: Drosophila SWR1 and NuA4 complexes are defined by DOMINO isoforms
Source: eLife. 2020 May 20;9:e56325. doi: 10.7554/eLife.56325 (PMC7239659; doi:10.7554/eLife.56325)
Supplement: Supplementary file 3. — Subunit composition of the yeast SWR1 and NuA4 were obtained from the manually-curated SGD database (https://www.yeastgenome.org) (CPX-2122 and CPX3155). For the human complexes, we refer to the EP400 complex subunits described in Dalvai et al., 2015 and to the SRCAP subunits described in Feng et al., 2018. [file elife-56325-supp3.docx]

| **SWR1.C** | | |
| --- | --- | --- |
| *D. melanogaster* | *S. cerevisiae* | *H. Sapiens* |
| DOM-B | Swr1 | SRCAP |
| BRD8 |  |  |
| MRGBP |  |  |
| YL-1 | Swc2 | YL-1 |
| GAS41 | Yaf9 | GAS41 |
| BAP55 | Arp4 | ACTL6a |
| ARP6 | Arp6 | ARP6 |
| DMAP1 | Swc4 | DMAP1 |
| MRG15 |  |  |
| PONT | Rvb1 | RUVBL1 |
| REPT | Rvb2 | RUVBL2 |
| HCF |  |  |
| PPS |  |  |
|  | Act1 | ACTIN |
|  | Swc3 |  |
|  | Swc5 |  |
|  | Swc7 |  |
|  | Vps71 |  |
|  | Vps72 |  |
|  | Bdf1 |  |
|  |  | ZNHIT1 |

| **NuA4.C** | | |
| --- | --- | --- |
| *D. melanogaster* | *S. cerevisiae* | *H. Sapiens* |
| DOM-A | Eaf1 | EP400 |
| BRD8 |  | BRD8 |
| MRGBP | Eaf7 | MRGBP |
| YL-1 | Swc2 | YL-1 |
| GAS41 | Yaf9 | GAS41 |
| BAP55 | Arp4 | ACTL6a |
| DMAP1 | Swc4 | DMAP1 |
| MRG15 | Eaf3 | MRG15 |
| *PONT* |  | RUVBL1 |
| *REPT* |  | RUVBL2 |
| ING3 | Yng2 | ING3 |
| TIP60 | Esa1 | KAT5 |
| E(Pc) | Epl1 | EPC1 |
| NIPPED-A | Tra1 | TRRAP |
| XBP-1 |  |  |
| CG12054 |  |  |
|  | Eaf6 | EAF6 |
|  | Act1 | ACTIN |
|  | Eaf5 |  |
